# Extracts from a Lecture “On the Present Position in Europe of Some of the Most Interesting and Important Points of Modern Surgery,” Recently Delivered as an Introductory Discourse

**Published:** 1852-02

**Authors:** Thomas D. Mütter

**Affiliations:** Professor of Surgery in Jefferson Medical College, Philadelphia


*Extracts from a Lecture "On the present position in Europe of some of the most interesting and important points of Modern Surgery," recently delivered as an Introductory Discourse.* By Thomas D. Mütter, M. D., Professor of Surgery in Jefferson Medical College, Philadelphia.

(Continued.)

*Opthalmic Surgery*, as I anticipated, from the numerous excellent and practical works, which from time to time have appeared from the teeming intellects of Lawrence, Mackenzie, Middlemore, Chelius, Elle, Vidal, Velpeau, Romig, Cullier, Roquetta, and others, I found in a most excellent condition. In truth, no department of our science appears to have been cultivated with more success, and that which, but a few years since, was "chaos and confusion dire," appears to have been touched with the wand of some mighty magician, and is now a bright and connected portraiture of nearly every disease to which the human eye is liable. I cannot, of course, attempt here a survey of the immense mass of novel as well as of useful information with which the science has been enriched by the labors of those to whom I have just referred.

*Ovariotomy.*—A distinguished philosopher, has classed man among the most cruel of animals; and certainly, were we to restrict our observations to the mere work of the surgeon, without entering into an investigation of the motives which lead him to the performance of bloody and terrific operations, the example alone, would be sufficient to lend countenance to the assertion, repugnant as it must be to the feelings of every one possessed of the common attribute of humanity. Certain it is, however, that some of our operations may be considered as supporting, to a limited degree, the charge made against our race; and there is none in the whole domain of Surgery better calculated to elicit, even among the profession, a more profound sensation of horror, or better deserves the epithet of cruel, than one recently introduced into practice; and were we not convinced that nothing but a fervent desire to relieve a suffering mortal, could induce a surgeon to undertake its performance, we should at once look upon its author as a being destitute of either sympathy or compassion, and richly deserving the detestation of his fellow men.

The operation to which I refer, is that for the removal of *ovarian tumors*, by what is called the *great incision.* In other words, by an incision that extends in a straight line from the *cartilago-ensiformis*, to the *symphisis pubis!* It is called the *great* or *major incision*, to distinguish it from another operation for the removal of diseased ovaria, in which the opening made into the abdomen extends but a few inches, and which was suggested by William Hunter, but has attained its present reputation in consequence, especially, of the labors of Jeaffreson.

As this subject is attracting a great deal of attention both abroad and at home, it will not be inapposite to furnish you with a slight sketch of its history and present position. It would appear that in consequence of the frequent failure of pure medical means, to relieve dropsy of the ovary, several surgical operations have from time to time been performed. Thus, some have advised "puncture of the cyst, evacuation of its contents, and then injection of some stimulating fluid, for the purpose of exciting adhesive inflammation;" others attempted a cure by making a free incision into the ovary, evacuating its contents and converting the opening into a fistulous sore, (Ledran, Houston, Voisin, &c.) Others again, suggested the removal of a part of the cyst, so as to make it empty its contents into the peritoneal sac, (Blundell, &c.) *Acupuncture*, with long needles, also has been performed, but the operation usually preferred has been simple *tapping.* Indeed, with the exception of the latter, all the others have been, with great wisdom abandoned, and the acknowledged failure of this operation to afford more than temporary relief in many cases, while in others it was followed by death, induced surgeons to seek for something upon which their confidence could, with greater security, be placed. Accordingly, we find that some fifty years since, L'Aumonier, of Rouen, extirpated an enlarged ovary, under the supposition that it was dropsical. The case turned out, however, to be one of *abscess* of the organ, and the patient ultimately recovered. This was unquestionably, I believe, the first removal of a diseased ovarium, but soon after, in 1809, Dr. McDowell, of Kentucky, performed the operation in a case of real ovarian dropsy, and the patient recovered. This successful result, induced others to repeat the experiment, and since that period a large number of cases have been reported, and undoubtedly others have been performed, of which no account has been furnished. But at no period, probably, has there existed so much excitement in reference to this operation, as at the present moment, and you will find, as is ever the case, when men allow feeling or interest to obtain a mastery over their judgment, that the most disgraceful acrimony and harshness of language has been indulged in toward each other, by the advocates, as well as the opponents of the measure in question. For my own part, I have endeavored faithfully and cautiously to examine the subject, being prejudiced neither for nor against it, and must confess, from the *information now furnished to the world*, I am induced to range myself among its opponents, except in cases of *unilocular cyst*, *without adhesions;* and even here I deem it altogether unjustifiable, until all other means have proved nugatory, and the fatal termination of the case without it appears inevitable. When had recourse to, it becomes the bounden duty of every surgeon to state candidly its dangers, and the probability of its failure. In order that my opinion may be borne out by sufficient reasons, I beg leave to offer a list of the most prominent objections urged by different authorities to the operation, and which must present themselves at once to every one who carefully investigates the merits of the question.

The objections to the operation, are

1st, The difficulty of arriving at a just diagnosis.

2d, The danger of the operation itself.

3rd, The nature of the disease does not sanction so violent a remedy.

4th, It is contended that palliatives will often succeed in rendering a patient comfortable during a long life.

5th, An operation does not always succeed in relieving a patient radically, even when she escapes the dangers immediately consequent to its performance.

6th, The disease may terminate spontaneously.

Such are the objections urged against *ovariotomy*, by the first surgeons of Europe, and I can with perfect truth declare that there is scarcely an eminent man abroad who lends the slightest countenance to the measure.

While we hope that future observations may divest the operation of many of its dangers, and establish a more correct diagnosis in the disease for the relief of which it has been proposed, we sincerely trust that no one will heedlessly attempt so hazardous a procedure, without duly reflecting upon the immense responsibility he assumes.

*Plastic Surgery.*—You will be anxious, I doubt not, to learn the estimation in which European Surgeons, generally, hold what is called *Plastic Surgery.* This department of our science, although in reality "old enough to speak for itself," may be considered a comparatively modern invention, for certainly the beautiful and perfect results attained in our time through its agency, far surpass anything that emanated from the hands of its original advocates and inventors, not excepting even the learned Taliacotius himself. These operations were for many years considered almost as fabulous, and have excited the ridicule of the wits of every age, including Butler, Voltaire and the polished Addison; and even now, notwithstanding the positive testimony of the first authorities in their favor, are supposed by many to be bare assertions destitute of truth, and as useless as they are apochryphal. But, gentlemen, both wit and opposition have been tried in vain, and the most distinguished men in Europe unite in awarding to the measure a high and commanding position among the most useful improvements of the age. When such authorities as Graefe,

Dieffenbach, Zeis, Chelius, Delpech, Dupuytren, Velpeau, Roux, Lisfranc, Larrey, Blandin, Labat, and Jobert, on the continent, and Brodie, Lawrence, Liston, Stanley, Fergusson, and others of high authority in England, declared their conviction of its utility, *Plastic Surgery* may be considered as having fought its battles, and will soon rest under the ægis of an established operation.

*Lithontripsy.*—One of the most striking characteristics of our nature is, that which leads us to doubt the value of every project or scheme originating with another. We cannot realize at once the fact, that some one else has discovered and brought to light something of which our own faculties have never taken cognizance ; and hence we admit its importance with hesitation, or boldly declare the statement of its advocates to be false, and contrary to reason or experience. Probably no operation in surgery more fully illustrates the correctness of these remarks, than *lithotrity* or *lithontripsy.* From the period of its introduction into practice by Leroy D'Etioles, Civiale, Heurteloup, and others, it has had to contend with fierce, violent, and most unjust opposition; and even down to the present moment, you will find surgeons decrying both the grinding and crushing processes, and declaring them to be, in the majority of cases, of no avail, while in others, they are positively murderous.

With the view of ascertaining the precise estimate placed upon the measure in Europe, I took especial pains to enquire of the surgeons in London and Paris, as to what was the real condition of the operation in their respective cities. In both I found it in high repute, but more especially was this the case in Paris. In the latter city, the dexterous and excellent surgeons, Civiale and Leroy d'Etioles, perform it almost daily; and while they acknowlege that *lithotomy* is still the operation best suited to many cases, they yet contend that it is far more dangerous, and gives rise to much more suffering than lithontripsy. This is certainly correct, and no one who gives the operation a fair trial, can hesitate for a moment to arrive at the same conclusion. No one contends that it is to supersede the use of the knife, but it is obvious that it must ere long be considered by far the safest and least painful mode of removing a stone from the bladder of an adult, unless the case be complicated with lesions of other organs in the vicinity. I may remark, that the original operation of

*lithotrity* has given place almost entirely to the more modern one of *lithontripsy.* Of *Liethectasy*, I heard but little, either in London or Paris, and the operation, though still recommended by some, cannot be considered as one at all popular with the profession at large.

*Hypodermatomy.*—No operation of modern times has attracted more attention, excited more controversy, been more shamefully abused, or unjustly lauded, than what has been termed by Sedillot, *Hypodermatomy* or *sub-cutaneous section*, and which has been, in some shape or other, so extensively employed for the relief of various deformities. As I have long been known as the advocate of this measure, when restricted to its proper limits, I made its investigation one of my principal objects, and it was with no little gratification, I assure you, that I found all operating surgeons, *without exception* I believe, while they reprobated its careless and indiscreet employment, declaring their entire confidence in the operation, when properly and judiciously practised. In short whenever I put the question, "What is your estimate of sub-cutaneous section in reference to deformities?" to any distinguished surgeon, either in England or on the continent, his answer was invariably this, "I consider it one of the greatest improvements in modern surgery, and cannot conceive that any surgeon who studies the result of the operation with care and fairness, can arrive at any other conclusion." Recollect this then, when you hear the method decried by those who have either never given it due attention, and are thus incompetent to decide upon its merits, or, who oppose it on what they consider correct principles, and are perfectly honest in this belief, (and I respect them for it,) or, who finally condemn it from prejudice alone. And rely upon it, that every surgeon abroad considers the various modifications of sub-cutaneous surgery, especial tenotomy, aponeurotomy, and myotomy, as the least dangerous, least painful and most useful, of all our means for the relief of deformities of various kinds.

*Stricture.*—A good deal of controversy is now going on in Europe in reference to the new treatment of stricture of the urethra by perineal section, proposed by Mr. Syme, of Edinburgh. That the operation may possibly prove useful in certain difficult cases of permanent stricture is possible, but that it can ever become a popular or necessary operation in the vast majority of cases of this disease, is scarcely probable. For the present, however, we are unable to speak decidedly upon this point, as the method has yet to be subjected to the test of observation and experience.

*Aneurism.*—You are all aware of the terrible nature of *aneurism*; one of the most fearful diseases to contemplate in its progress, as well as one of the most difficult to manage with success. The danger attendant upon the usual operation for this affection, and the failure of the old method of treatment by *compression*, induced, a few years since, Mr. Hutton, of Dublin, and afterwards Drs. Cusack, Bellingham, Kirby and Harrison, of the same city, to attempt its cure by a new application of compression, called by them *intermittent* or *irregular*. I found surgeons very much divided in opinion relative to the merits of this new measure, and several very unfortunate cases in which it was employed, were recently announced in the London Hospitals. From my own observations, however, and from what I could gather from some of the most experienced of those who had employed it, I am led to consider the plan one of the most important improvements in modern surgery. It can never entirely supersede the use of the ligature, but it will answer in a great majority of cases.

*Parotid Gland.*—As extirpation of the *Parotid Gland* has given rise to much controversy on this side of the Atlantic, I was anxious to ascertain the estimate placed upon the measure by surgeons abroad; and therefore made it a subject of diligent enquiry. As I anticipated, their exists great contrariety of opinion in relation to the *utility* of the process, but I found *none* who doubted its *possibility*. Indeed, the question seemed to bear almost *exclusively* upon the first proposition.

(To be continued.)

